# Impact on Renal Function and Hospital Outcomes of an Individualized Management of Cardiopulmonary Bypass in Congenital Heart Surgery: A Pilot Study

**DOI:** 10.1007/s00246-021-02680-4

**Published:** 2021-07-22

**Authors:** Riccardo Giuseppe Abbasciano, Stiljan Hoxha, Dania Gaburro, Siliva Surdo, Tiziano Menon, Leonardo Gottin, Giuseppe Faggian, Giovanni Battista Luciani

**Affiliations:** 1 Department of Cardiovascular Sciences, University of Leicester, Leicester, UK; 2 Section of Cardiac Surgery, Department of Surgery, Dentistry, Pediatrics and Gynecology, University of Verona, O. C. M. Piazzale Stefani 1, 37126 Verona, Italy; 3 Section of Anesthesiology, Department of Surgery, Dentistry, Pediatrics and Gynecology, University of Verona, Verona, Italy

**Keywords:** Congenital heart diseases, Cardiac surgery, Cardiopulmonary bypass, Acute kidney injury, Organ protection

## Abstract

During cardiopulmonary bypass (CPB), high flows can allow an adequate perfusion to kidneys, but, on the other hand, they could cause emboli production, increased vascular pressure, and a more intense inflammatory response, which are in turn causes of renal damage. Along with demographic variables, other intra-operative management and post-operative events, this might lead to Acute kidney injury (AKI) in infants undergoing cardiac surgery. The aim of our study was to investigate if a CPB strategy with flow requirements based on monitoring of continuous metabolic and hemodynamic parameters could have an impact on outcomes, with a focus on renal damage. Thirty-four consecutive infants and young children undergoing surgery requiring CPB, comparable as for demographic and patho-physiological profile, were included. In Group A, 16 patients underwent, for a variable period of 20 min, CPB aiming for the minimal flow that could maintain values of MVO_2_ > 70% and frontal NIRS (both left and right) > 45%, and renal NIRS > 65%. In Group B, 18 patients underwent nominal flows CPB. Tapered CPB allowed for a mean reduction of flows of 34%. No difference in terms of blood-gas analysis, spectroscopy trend, laboratory analyses, and hospital outcome were recorded. In patients developing AKI (20%), renal damage was correlated with demographic characteristics and with renal NIRS during the first 6 h in the ICU. A safe individualized strategy for conduction of CPB, which allows significant flow reduction while maintaining normal hemodynamic and metabolic parameters, does not impact on renal function and hospital outcomes.

## Introduction

Acute kidney injury (AKI) is one of the most common complication after congenital cardiac surgery, occurring in 6 to 50% patients, with an associated mortality ranging from 20 to 61%, based on the different diagnostic criteria adopted and the characteristics of the population studied [1]. Therefore, management of renal perfusion is crucial during CPB, and a decreased renal flow despite ideal hemodynamic management can lead to acute tubular necrosis which in turn may lead to AKI [2].

Historically, cardiopulmonary bypass (CPB) flows during cardiac surgery have been calculated solely on patient weight. However, as a result, CPB flows employed for pediatric patients undergoing surgery for congenital cardiac diseases are often arguably overestimated. Higher CPB flows are related to increased risk of morbidity (hemodilution, higher need for transfusion, oedema, Systemic inflammatory response syndrome (SIRS), and a more conspicuous volume of warm venous blood returning to the operating field, which in turn can hinder both surgeon’s vision and myocardial protection). Nonetheless, decreasing flows could lead to inadequate perfusion, possibly resulting in organ damage (e.g., acute kidney injury, neurological morbidity) with prolonged and more complicated post-operative length of stay.

We hypothesized that a different strategy of CPB management with tapered flows, tailored on patient’s metabolic needs measured by means of near-infrared spectroscopy (NIRS) and continuous in-line blood-gas monitoring, could help reducing detrimentally high flows safely, while monitoring end-organs necessities in real time during surgery.

The aim of this work is to assess an approach, previously investigated [3], in terms of its effects on renal function, defined according to KDIGO criteria [4] and to explore whether AKI incidence in a pediatric population undergoing cardiac surgery for congenital heart disease in our Center can be explained by pre-operative and intra-operative characteristics.

## Patients and Methods

### Patients

Approval for this study was granted by the institutional review board, while the families of patients qualifying for the study were asked for consent.

Between May 2016 and October 2017, families of all consecutive infants and young children undergoing elective surgical repair using CPB were offered to participate in the study. A total of 34 patients’ families consented (Table 1). Patients with a body weight over 15 kg, patients whose surgery required deep hypothermia (< 22 °C), patients with preoperative renal dysfunction, and patients who were treated with an emergency surgical procedure were excluded from our study.

**Table 1:** Demographics and intra-operative data

	Tapered CPB	Standard CPB	Total	*P*-val
Patients number	16	18	34	
Male/Female ratio	11\5	6\12	17\17	
Age (days, Mean ± SD)	177 ± 221	185 ± 184	181 ± 199	0.914
Age range (days)	39–825	5–557	5–825	
Weight (kg, Mean ± SD)	5.3 ± 1.6	5.3 ± 2.3	5.3 ± 1.9	0.514
Weight range (kg)	2.9–9.6	2.7–10	2.7–10	
Height (cm, Mean ± SD)	59.3 ± 8.5	61.7 ± 11.9	60.6 ± 10.2	0.497
Height range (cm)	45–84	49–87	45–87	
BSA (m^2^, Mean ± SD)	0.29 ± 0.06	0.29 ± 0.09	0.29 ± 0.10	0.921
BSA range (m^2^)	0.19–0.47	0.18–0.47	0.18–0.47	
Nominal flow (cc/min, Mean ± SD)	814 ± 205	807 ± 302	810 ± 253	0.943
CPB duration (min, Mean ± SD)	106 ± 35	125 ± 92	116 ± 71	0.453
Cross clamping (min, Mean ± SD)	59 ± 27	59 ± 56	59 ± 44	0.981

*CPB* cardiopulmonary bypass, *SD* standard deviation

In group A, CPB was managed indexing flows to the minimum value to maintain SvO_2_ > 70%, while checking frontal rSO_2_ to be higher than 45% and renal rSO_2_ to be higher than 65% for a period of 20 min, 10 min after aortic cross-clamping (tapered flows). In group B, CPB was conducted according to conventional flow parameters.

**Fig. 1:**
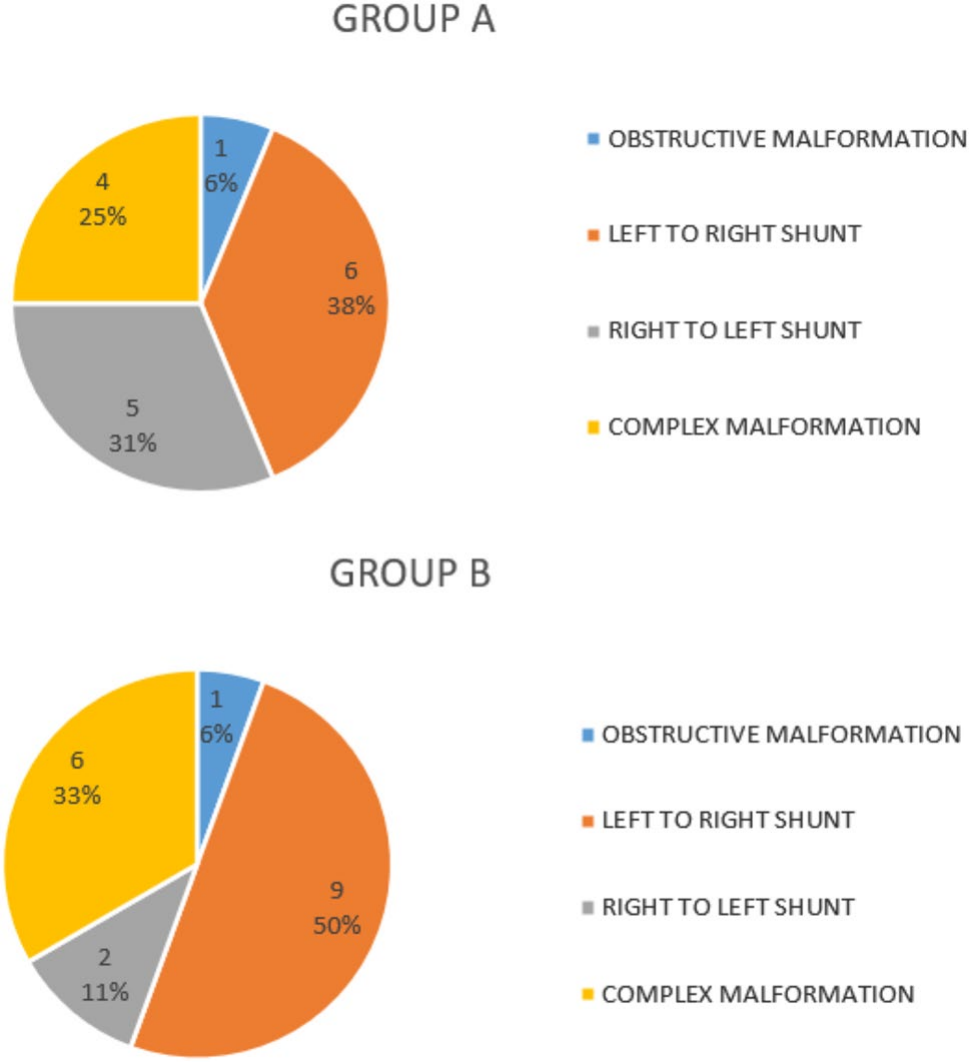
Physio-pathological classification. Study cohort composition in terms of the physio-pathology of the underlying conditions

Blood-gas data from CDI and CPB parameters were collected. NIRS (left frontal, right frontal, and renal) were registered before the operation, throughout surgery, and for a minimum of 24 h in the Intensive care unit (ICU). The pediatric-sized sensor to measure renal oximetry on the right flank overlying the right kidney were placed on arrival in the operating room, along with two frontal sensors. Demographic (age, weight, height) and laboratory (CRP, pCT, creatinine, WBC) data were collected. Risk adjustment for congenital heart surgery (RACHS-1) classification [5] was used to assess the surgical complexity of all participants (Fig. 1).

### CPB Methods

Choice of oxygenator was based on flow requirements: Capiox® FX05 (Terumo Cardiovascular Systems Corporation, Ann Arbor, MI) if nominal flow was lower than 1500 mL/min, with perfusion tubing system Terumo x-Coating ® (Terumo Cardiovascular Systems Corporation, Ann Arbor, MI) with integrated arterial filter or Dideco® Kids D101 (Sorin Group, Mirandola, Modena, Italy) if nominal flow was greater than 1500 mL/min, with perfusion tubing system Phisio and arterial filter D131 (Sorin Group, Mirandola, Modena, Italy). Tubing prime was composed by ringer-acetate, bicarbonate, and albumin; mannitol was added if necessary. Blood was added only after Hb concentration and patient weight evaluation (blood was added in patients weighing less than 10 kg). During CPB, threshold level to add packed red blood cells to prime was hemoglobin lower than 8.5 g/dL. Vacuum assisted (up to − 25 mmHg) venous drainage use and position of the oxygenator support closest to the operative table thereby reducing tubing length, were also instrumental to further reduce prime volume. Under normothermic conditions (nasopharyngeal temperature 34–36 °C), flow was maintained between 120 and 150 ml/kg/min. Cardioplegic arrest was induced by anterograde Buckberg cardioplegia, administering type A solution at induction (15 ml/kg/min), B1 maintenance solution every 20 min, at a temperature between 4 °C and 10 °C, and normothermic blood as a reperfusion solution before clamp release. Modified ultrafiltration was routinely applied before removal of arterial and venous cannulae.

### Metabolic Monitoring

Terumo CDI 500® (Terumo Cardiovascular Systems Corporation, Ann Arbor, MI) was used for continuous in-line blood-gas monitoring. This system updates data every 5 s. Those data were collected into an electronic chart every 30 s, from bypass start to end. CDI was calibrated by analysing venous blood samples at bypass start and rechecked every 20 min during CPB. The assessment of cerebral metabolism was conducted by continuous rSO2 measurement from both cerebral hemispheres and renal region using NIRS technology (EQUANOX Model 7600 Regional Oximetry System, Nonin Medical, Inc. Plymouth, Minnesota) throughout the entire operation, recording data electronically every 5 min. We focused the analysis of our data on five distinct time frames (T0: CDI calibration, T1: aortic cross-clamp, T2: 5 min after the start of metabolic CPB management, T3: of end of metabolic CPB management, T4: after clamp removal). Patients in Group B did not undergo the 20 min period of metabolic CPB management, therefore no T2 and T3 period was considered.

### Statistical Analysis

Continuous variables are presented as mean value ± standard deviation (SD) and categorical variables as a number or a percentage. Normal distribution of all collected data was ensured before analysis. Statistical analysis was performed using the software SPSS for Windows version 22 (IBM, Chicago, Illinois). Continuous variables with normal distribution were compared using a Student t-test, for variables without normal distribution, a Mann–Whithney U-test was employed; for categorical values Fischer’s Exact Test was employed. A multivariate logistic regression analysis was conducted to identify a potential independent predictor for AKI among the pre-operative and intra-operative variables. Moreover, a linear regression analysis was performed to investigate for post-operative period trends for NIRS values and laboratory parameters. For all the tests, a *P*-value of ≤ 0.05 was deemed statistically significant. Descriptive statistics illustrate post-operative morbidity.

## Results

The proposed metabolic management of CPB allowed an overall average tapering of flows of 34%. As expected, reduction was greater in the hypothermic population (47%) and slightly less among normothermic participants (30%) (Table 2).

**Table 2:** Flow reduction in metabolic management CPB

	Normothermia	Hypothermia	Whole population
Nominal flow (ml/min)	812 ± 221	820 ± 102	814 ± 198
Tapered flow (ml/min)	574 ± 233	424 ± 50	537 ± 213
Flow reduction (%)	− 29.6 ± 13.5	− 47 ± 12.1	− 34.4 ± 15.0

*CPB* cardiopulmonary bypass

When pre-operative characteristics were compared between the two groups (Table 3), no significant difference was recorded. Group A and Group B did not show any significant difference in terms of intraoperative variables (Table 4). Even if flow reduction from the nominal value was significant, no difference was reported in terms of average flow maintained during CPB or the lowest value of flow reached during surgery. Moreover, CPB time and aortic cross-clamping times were comparable between the two groups.

**Table 3:** Comparison of pre-operative characteristics between the two groups

Pre-operative	Tapered flows	Mean	Std. deviation	*P*
RACHS1	Yes	2.88	1.20	0.424
	No	2.56	1.10	
Age (days)	Yes	177.63	220.72	0.919
	No	184.72	185.01	
Weight (kg)	Yes	5.31	1.58	0.981
	No	5.32	2.29	
Length (cm)	Yes	59.25	8.53	0.489
	No	61.72	11.93	
BSA (m^2^)	Yes	0.29	0.06	0.895
	No	0.29	0.09	
SvO_2_ (%)	Yes	69.75	3.44	0.438
	No	71.89	10.89	
Frontal NIRS (Left) (%)	Yes	63.88	8.29	0.857
	No	64.50	11.67	
Frontal NIRS (Right) (%)	Yes	62.06	8.91	0.390
	No	65.22	12.13	
Renal NIRS (%)	Yes	76.56	9.30	0.949
	No	76.39	6.24	

*NIRS* near-infrared spectroscopy, *RACHS* risk adjustment for congenital heart surgery

**Table 4:** Comparison of intra-operative characteristics between the two groups

Intra-operative	Tapered flows	Mean	Std. deviation	*P*
CPB temperature (C°)	Yes	31.69	3.00	0.820
	No	31.39	4.14	
CPB duration (min)	Yes	106.81	34.40	0.435
	No	125.39	92.24	
Cross clamping duration (min)	Yes	59.81	26.90	0.980
	No	59.44	56.02	
Nominal flow (ml/min)	Yes	814.13	204.92	0.943
	No	807.61	302.35	
Mean arterial pressure (mmHg)	Yes	51.50	6.22	0.201
	No	48.50	7.07	
Nadir_flow (ml/min)	Yes	411.63	150.87	0.180
	No	548.67	388.64	
Average_flow (ml/min)	Yes	556.36	172.41	0.265
	No	676.22	405.77	

*CPB* cardiopulmonary bypass

Data analysis obtained from the CDI during CPB in group A proved only pCO_2_ to be significantly higher at the end of the 20 min period. No other parameter, including lactates, was significantly modified by the test (Table 5).

**Table 5:** CDI values in group A during CPB

		T0	T1	T2	T3	T4	P (T1–T3)
pH		7.46 ± 0.09	7.46 ± 0.14	7.45 ± 0.08	7.39 ± 0.07	7.39 ± 0.09	NS
pCO_2_	(mmHg)	31.0 ± 7.0	30.1 ± 6.1	32.0 ± 5.2	36.9 ± 5.2	37.1 ± 5.5	< 0.05
pO_2_	(mmHg)	270 ± 81	261 ± 48	233 ± 43	233 ± 47	229 ± 52	NS
BE	(mmol/L)	− 2.5 ± 6.1	− 1.9 ± 4.1	− 0.7 ± 2.6	− 0.9 ± 2.5	− 1.3 ± 3.7	NS
SO_2_	(%)	98.8 ± 1.3	98.9 ± 0.3	98.8 ± 0.5	98.6 ± 0.8	98.6 ± 0.9	NS
Hb	(g/dL)	8.4 ± 1.1	8.2 ± 1.0	8.4 ± 1.1	8.8 ± 1.2	8.2 ± 0.8	NS
SvO_2_	(%)	73.2 ± 6.8	71.8 ± 5.0	70.3 ± 1.3	69.9 ± 1.7	64.4 ± 5.1	NS
Q	(ml/min)	726 ± 182	636 ± 201	516 ± 232	550 ± 202	714 ± 240	NS
VO_2_	(ml/min/m2)	65.5 ± 19.6	63.4 ± 20.8	57.8 ± 19.7	63.4 ± 22.5	89.6 ± 30.3	NS
DO_2_	(ml/min/m2)	299.4 ± 48.4	252.7 ± 54.1	224.5 ± 60.9	245.5 ± 65.3	278.3 ± 76.2	NS
LACT	(mmol/L)	1.8 ± 0.7	1.6 ± 0.6	1.5 ± 0.7	1.7 ± 0.8	2.1 ± 0.9	NS
NIRS R	(%)	65 ± 8	63 ± 9	60 ± 9	60 ± 9	55 ± 12	NS
NIRS L	(%)	64 ± 9	62 ± 10	58 ± 9	59 ± 10	55 ± 9	NS
NIRS K	(%)	77 ± 8	79 ± 8	80 ± 7	80 ± 6	79 ± 6	NS

*CPB* cardiopulmonary bypass, *LACT* Lactates, *NIRS* near-infrared spectroscopy, *R* right frontal, *L* left frontal, *K* Renal

**Fig. 2:**
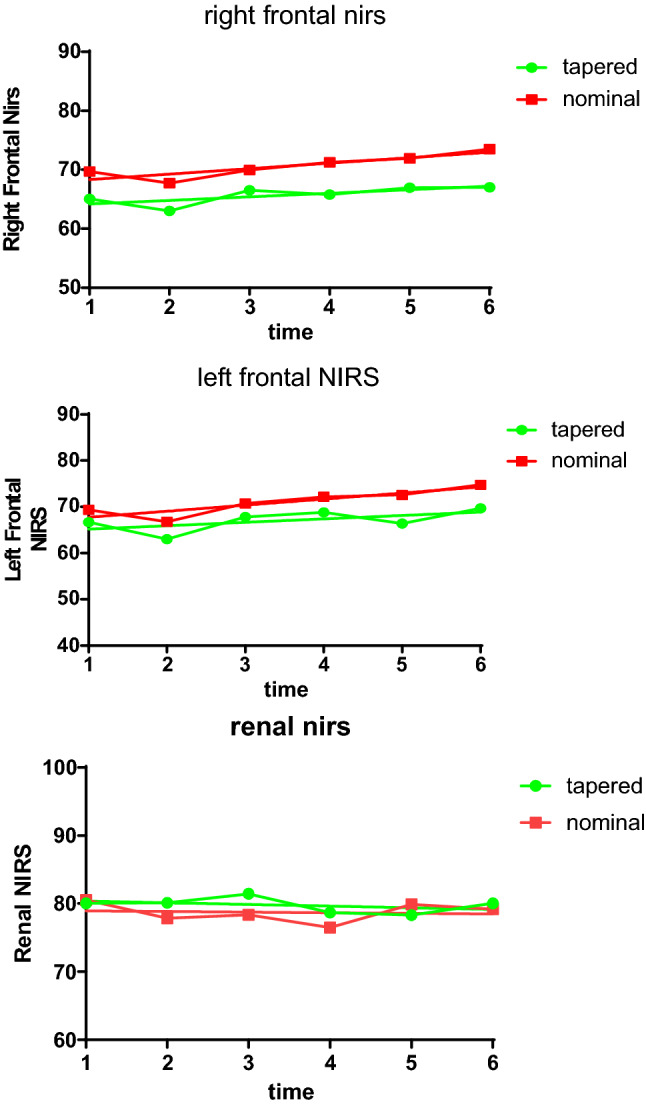
NIRS trend. Mean Right Frontal, Left Frontal, and Renal Near-infrared Spectroscopy values recorded at different study phases (1) pre-operatively; (2) during CPB; (3) ITU admission; (4) 12 h after admission in ITU; (5) 18 h after admission in ITU; (6) 24 h after admission in ITU

**Fig. 3:**
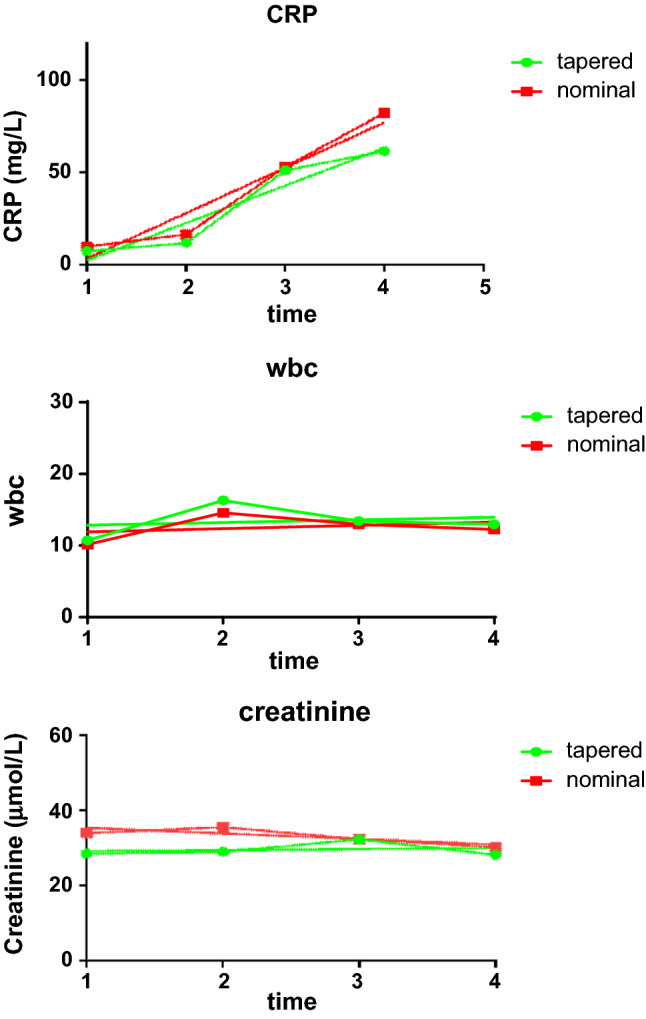
Inflammatory markers and Creatinine trend. Mean C-reactive protein, White blood cell count and Creatinine trend recorded at (1) ITU admission; (2) First post-operative day; (3) second post-operative day; (4) third post-operative day

Post-operative NIRS trend was compared among group A and group B (Fig. 2). No significant difference was reported at any time point. When comparing post-operative laboratory values (Fig. 3), no significant difference, in both inflammatory indexes and markers of renal function, was found. PCT was excluded from the analysis because of the high number of missing values.

Seven cases of AKI were identified (3 in group A, 4 in group B). Only the KDIGO criteria for serum creatinine change were used (at least 1.5 times baseline or at least 26.5 µmol/l increase), since liberal use of diuretics could have otherwise biased our analysis. Comparison between patients presenting AKI with the unaffected ones allowed to identify some significant differences regarding, mostly, demographic parameters (Table 6). During surgery, average flows were significantly lower in patients presenting post-operative AKI. From the analysis of post-operative course, renal NIRS 6 h after the intervention were significantly lower (mean difference 8.5; *p* value 0.035) in patients that would develop AKI. No other NIRS value was significantly altered at any of the time points of our study. Moreover, aside from the expected significant increase in creatinine values, no other laboratory parameter measured was different between patients affected by AKI and unaffected ones.

**Table 6:** Comparison of pre-operative and intra-operative characteristics between patients incurring in AKI and unaffected patients

Pre-operative	AKI	Mean	Std. deviation	*P*
RACHS1	Yes	2.71	0.95	0.98
	No	2.70	1.20	
Age (days)	Yes	61.86	45.37	0.02
	No	212.37	212.56	
Weight (kg)	Yes	4.32	1.12	0.14
	No	5.57	2.06	
Length (cm)	Yes	54.23	4.34	0.007
	No	62.19	10.92	
BSA (m^2^)	Yes	0.25	0.47	0.116
	No	0.30	0.83	
SvO_2_ (%)	Yes	75.29	8.95	0.113
	No	69.74	7.78	
Frontal NIRS (Left) (%)	Yes	59.29	5.31	0.150
	No	68.50	10.68	
Frontal NIRS (Right) (%)	Yes	57.57	11.47	0.087
	No	65.33	10.10	
Renal NIRS (%)	Yes	76.86	5.75	0.884
	No	76.37	8.22	
CPB duration (min)	Yes	143.29	18.99	0.270
	No	109.74	77.70	
Cross clamping duration (min)	Yes	66.29	30.97	0.661
	No	57.89	47.30	
Mean arterial pressure (mmHg)	Yes	47.57	5.623	0.331
	No	50.52	6.980	
Average flow (ml/min)	Yes	455.86	120.678	0.015
	No	662.33	342.067	
Nadir flow (ml/min)	Yes	341.00	113.647	0.167
	No	521.30	328.747	

*CPB* cardiopulmonary bypass, *NIRS* near-infrared spectroscopy, *RACHS* risk adjustment for congenital heart surgery

In a logistic regression, among the pre-operative characteristics, NIRS measurements and perfusion parameters, only renal NIRS 6 h after surgery were a significant independent predictor for developing renal injury (AIC 32.48, *p* value 0.043). When the parameters that performed better (NIRS 6 h after surgery, height, and pre-operative creatinine) were included in a multiple regression model, the model returned an AIC of 25.88 and consequently a p value of 0.001.

**Fig. 4:**
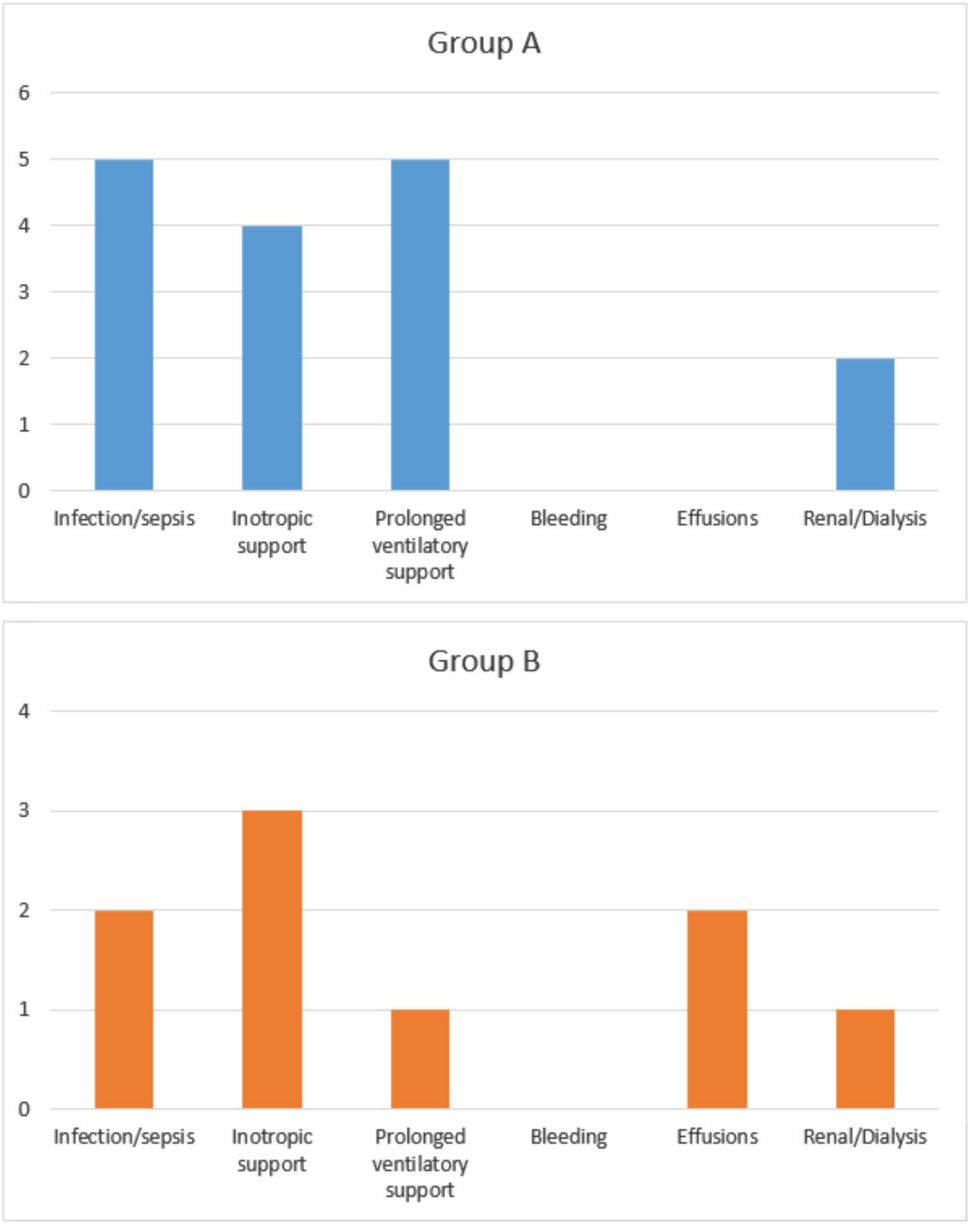
Hospital outcomes. Summary of post-operative complications in the two study cohorts

Clinical outcome was evaluated. Overall hospital morbidity was comparable to what normally expected for open-heart surgery in the infant and pre-school child age range, and it was similar between the two groups. AKI incidence was 20%, lower than most of data reported in literature. Five patients (4 group A; 4 group B) required a pharmacological inotropic support higher than 0.05 mcg/Kg/min longer than 24 h, two patients needed mechanical life support (ECMO; one patient per group), eight patients needed prolonged (greater than 96 h) mechanical respiratory support (5 group A; 1 group B), seven manifested organ infection (5 group A, 2 group B), requiring prolonged (14 days) antibiotic therapy, two patients presented recurrent pleural effusions (group B), three patients required dialysis (2 group A; 1 group B), and no patient needed re-exploration for bleeding (Fig. 4). No neurological complication was observed. No mortality was registered during the present study.

## Discussion

The brain and the kidney may be considered the two opposite poles of regional circulation [6]. In fact, while the brain benefits from a somehow constant blood flow by mechanism of autoregulation (only minimally influenced by sympathetic control) to sustain its high metabolic demand and therefore its high oxygen extraction ratio, the kidney normally receives a high regional flow, but has a low oxygen extraction rate and undergoes intense regulation by the sympathetic system.

Pediatric patients undergoing CPB for congenital heart diseases constitute a fragile population due to the immaturity of their organs and their potential to develop serious damages with even the slightest change of perfusion parameters. Although neurological consequences must always be a main concern for the surgeon and the perfusionist, kidneys should be considered among the organs most susceptible to the risk of inadequate perfusion, too. Moreover, their physiology, less protected by autoregulation when compared to the brain, allows a more immediate assessment of the consequences of the perfusion management.

Study of renal function allowed us to further refine a theme already investigated in a previous study [3], applying it to the specific setting: whether maintaining relatively high flows can be protective against AKI or risks related to unnecessary high flows (such as inflammatory response or higher rate of emboli) may on the contrary be detrimental to the prevention of renal damage. While there is a copious amount of works exploring non-invasive monitoring of pediatric patients during CPB and in the ICU [1, 7–14], most of them are focused on a single phase of these settings. Moreover, to our knowledge, no work has previously investigated the effects of a safe flow reduction on the occurrence of AKI. Therefore, in order to investigate if a strategy of tapered flows CPB was beneficial to renal protection we planned a study to compare spectroscopy trends, laboratory parameters, and clinical outcomes in a population of children undergoing CPB, comparing traditional management (nominal flows) and metabolic management (tapered flows).

Different biomarkers (e.g., Cystatin C and NGAL) are being considered for the early detection of renal injury. Nonetheless, it has been shown how renal NIRS recorded intraoperatively may perform better than said biomarker in detecting AKI [1]. Our results are in line with the findings by Hazle et al.: although renal NIRS recorded during CPB were not significantly different in patients developing AKI and in unaffected ones, a significant difference was reported 6 h after surgery, before any alteration in serum creatinine became manifest. This may suggest a critical role for NIRS monitoring since it would grant the time to enact necessary countermeasures to the caregiver.

The efficacy of near-infrared spectroscopy is nowadays widely appreciated in everyday practice in adult cardiac surgery, since the first reporting of this methodology by Jobsis [15] in 1977 and the first description of clinical studies by Ferrari and colleagues in 1985 [16]. The great potential of extending the use of this technology in pediatric cardiac surgery lies in its working principle itself. NIRS evaluate real-time regional oximetry by assessing the different absorption of near-infrared wavelengths by oxygenated and deoxygenated hemoglobin thanks to a transmitter and a receiving optode placed reached by photons traveling an elliptical path, therefore a small skin organ distance such as the one in children with low body weight becomes a great benefit when using this technique.

In the presented experience, frontal NIRS and renal NIRS measurements were combined. In fact, as Gil Anton and colleagues showed combined NIRS correlate better with hemodynamic measurement (such as ScvO_2_), therefore offering a higher prognostic value [17]. The advantages of NIRS measurement in different body parts, aside from reducing biases coming from cerebral blood flow and oxygen supply autoregulation in low cardiac output states [7] may be represented by the different information renal NIRS can provide, not necessarily related to hemodynamic condition, such as the detection of compartmental syndrome and the consequent indication to drainage, as shown by Di Nardo and colleagues [18]. Moreover, although as Ranucci and colleagues have shown [19], NIRS correlates with superior vena cava oxygenation in children undergoing pediatric cardiac surgery, we agree with Samraj and colleagues by stating the necessity to assess both regional saturation by spectroscopy and venous oxygen saturation [20]. In fact, while regional spectrometry assesses local tissue oxygen delivery and oxygen consumption, parameters like ScvO_2_ can evaluate these data in the whole body. Moreover, ScvO_2_ allows to overcome some intrinsic limitations of NIRS monitoring, such as inter- and intra- individual variability, falsely low values secondary to agitation or to regional anomalies (e.g., scars) and the absence of threshold for normative data.

Results from our study confirm previous findings [3] by showing a comparable outcome in terms of laboratory trends and morbidity between group A and group B; moreover, from the analysis of patients developing AKI, we proved how this complication in our population seems unrelated to CPB management and on the other hand associated to patient demographic characteristics. Although lower flows were recorded in the cohort that developed AKI, these values were not significant independent predictors of renal injury. In fact, flow is ultimately derived from patients’ demographics, which on the other hand were important predictor of AKI in the multiple regression model.

Our results seem to suggest also that the immediate post-operative (renal NIRS alteration 6 h after CPB) period plays a major role in the genesis of renal injury, at least on par with the operative period, since no significant difference in blood-gas analysis and spectroscopy data between the two groups despite the different CPB management strategy could be identified. The role of renal spectroscopy in the timely diagnosis of AKI is particularly promising, as supported by similar studies [21], in which NIRS outperformed functional assessment (creatinine) alone in diagnosing important subclinical organ damage.

### Limitations

Limitations to our work comes from the small sample considered and the intrinsic heterogeneity of conditions in our population; however, we believe that tighter exclusion criteria would have been detrimental to the analysis of outcomes by hindering the comparison with the ordinary population treated in our Unit.

No urinary biomarker was employed in our investigations; nonetheless these are not yet considered in KDIGO guidelines and, once again, our aim was to perform an analysis that could be close to the clinical routine of laboratory exams.

Further multicenter studies considering a higher number of patients would be highly beneficial to assess outcomes and would prove useful to cost–benefit analysis concerning NIRS employ, a matter as of today, yet to be investigated.

## Abbreviations

AKI: Acute kidney injury
CPB: Cardiopulmonary bypass
CRP: C-reactive protein
ICU: Intensive care unit
NGAL: Neutrophil gelatinase associated lipocalin
NIRS: Near-infrared spectroscopy
PCT: Procalcitonin
RACHS-1: Risk adjustment for congenital heart surgery
SIRS: Systemic inflammatory response syndrome
WBC: White blood cell
